# The Prevalence of and Factors Associated with Alcohol-Related Problems in a Community Sample of African American Women

**DOI:** 10.1155/2016/7513827

**Published:** 2016-09-26

**Authors:** Hugh Klein, Claire E. Sterk, Kirk W. Elifson

**Affiliations:** ^1^Kensington Research Institute, Silver Spring, MD, USA; ^2^Rollins School of Public Health, Emory University, Atlanta, GA, USA

## Abstract

*Purpose*. This study examines the prevalence of alcohol-related problems, the factors underlying these problems, and whether or not there is evidence of syndemic effects in a community population of southern, urban African American women.* Methods*. Questionnaire-based interviews were conducted with 817 women, all African American, from 80 targeted census block groups in Atlanta, Georgia.* Results*. Most of the alcohol users (67.8%) experienced at least one problem as a result of their alcohol (ab)use, with most women experiencing two or more such problems. Eight factors were found to be associated with experiencing more alcohol problems: being aged 30 or older, having had no recent health insurance, lower levels of educational attainment, self-identifying as lesbian or bisexual, experiencing greater amounts of childhood maltreatment, greater impulsivity, perceiving one's local community or neighborhood to be unsafe, and having a larger number of criminally involved friends.* Conclusions*. Drinking-related problems were prevalent in this population. Numerous factors underlie the extent to which African American women experienced problems resulting from their alcohol use. There is strong evidence of syndemic-type effects influencing drinking problems in this population, and future efforts to reduce the negative impact of alcohol (ab)use ought to consider the adoption of programs using a syndemics' theory approach.

## 1. Introduction

Nearly two-thirds of American women aged 18 or older (66.5%) report having consumed alcohol at least once during the preceding year, with nearly two-thirds of those women (i.e., 49.7% of all adult women) reporting having had a drink during the previous month [1]. African Americans are somewhat less likely than persons of other racial groups to report having used alcohol during the preceding year (62.3% versus 72.0%) or during the preceding month (47.3% versus 57.7%) [1]. Despite being less prevalent than in the US population at large, particularly when compared with their Caucasian counterparts [2], both alcohol use and abuse are commonplace among African American women. Among alcohol-using adults, however, a slightly larger proportion of African Americans may be defined as binge alcohol users (46.3% of African American drinkers versus 43.7% of drinkers of other races) [1]—a finding that has been mirrored in other research as well [3]. Moreover, approximately 7.7% of African American adults—comparable to the percentage in the United States as a whole—meet the diagnostic criteria for alcohol abuse or alcohol dependence [1].

Research examining the factors associated with and/or underlying alcohol abuse among African American women in community samples, as opposed to clinical or criminal justice populations, has been limited. The available research has identified several possible influences on the drinking practices and problems of members of this population. For example, based on their study of African American women living in a rural area, Boyd et al. [4] reported that the best predictors of alcohol problems were experiencing childhood maltreatment experiences, violence and/or being victimized in adulthood, reliance upon alcohol in order to escape from one's problems and stresses, and reliance upon alcohol in order to cope with one's problems and stresses. Caetano et al. [5] found that, compared to Caucasian and Latina women, African American women are more likely to consume alcohol in what the authors called “hazardous situations,” such as driving after drinking. Relying upon their large-scale study of California adults aged 60 or older, Bryant and Kim [3] noted that being a cigarette smoker was a risk factor for binge drinking, particularly among African American women. The present authors consider it noteworthy for the current paper that previously published works have not addressed the relevance of neighborhood factors (e.g., perceived safety of one's neighborhood of residence, neighborhood cohesion) or personal support factors (e.g., number of male or female friends, type and extent of personal relationships with friends/acquaintances) to the pervasiveness of alcohol-related problems among African American women, nor has ample attention been devoted heretofore to such factors as psychological functioning or socioeconomic characteristics as they pertain to drinking problems in this population.

In the present paper, which is based on a community sample of African American adult women residing in the South, we address the following research questions: (1) How prevalent are alcohol use and drinking-related problems in this population? (2) What factors are associated with incurring a larger number of alcohol-related problems? (3) Is there evidence of potentiating, or syndemic, effects among the key predictors of drinking problems in this population?

**Figure 1:**
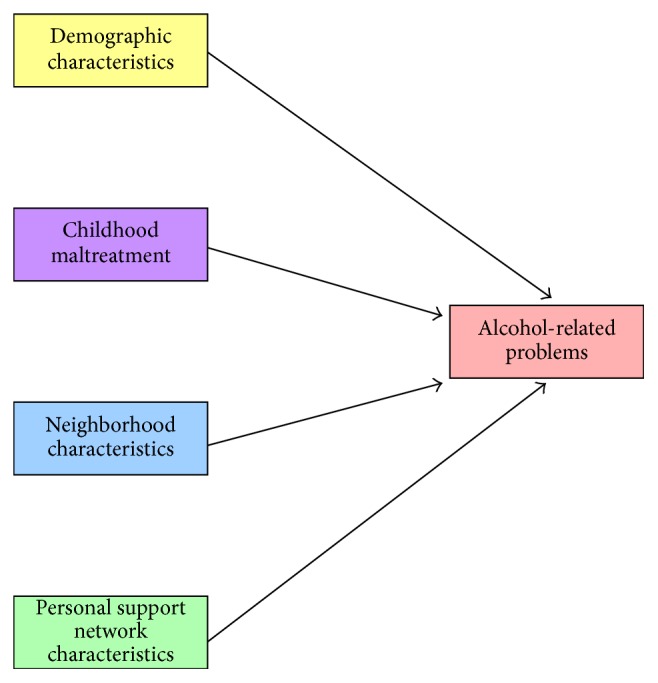
Conceptual model.

Regarding the latter question, for readers who may be unfamiliar with the construct of syndemic effects, we encourage consultation with Singer's [6] work. Basically, one of the main tenets of syndemics theory is that certain types of influences cooccur in such a manner so as to heighten the effects of one another, thereby making adverse health outcomes even more severe than they would have been had these effects not cooccurred. In the syndemics theory-based conceptual model used in the present research (see Figure 1), four types of measures are hypothesized to interact syndemically, such that they influence one another and the main alcohol-related problems outcome measure. These are (1) sociodemographic characteristics, (2) childhood maltreatment experiences, (3) neighborhood characteristics, and (4) personal support network characteristics. Our hypothesis is that these various factors will operate both individually and collectively/interactively to affect the risk of experiencing a greater number of alcohol-related problems.

In other studies, syndemics theory has been applied to study a wide array of health behaviors among a variety of populations within the United States [7–11]. Very rarely, if ever, though, have these studies been conducted with noninstitutionalized, community samples of women, particularly African American women. Moreover, although it is not at all uncommon for syndemics-focused studies to incorporate substance use/abuse measures into their models (see, e.g., [12–14]), almost all of these studies emphasized illegal drug use/abuse or polydrug abuse rather than alcohol use/abuse specifically and separately from other types of substance use/abuse. The authors would like to point out that there has been some syndemic theory-focused research based on populations outside of the United States, in which alcohol specifically was the focus rather than substance use/abuse more generally; examples include Hatcher et al.'s [15] study of South African men, Pitpitan and colleagues' [16] study of South African women, and Padilla et al.'s [17] work with tourism employees in the Dominican Republic. By addressing this very issue, the present study can make an important contribution to the scientific literature.

## 2. Methods

### 2.1. Procedures

Data for this study were collected as part of People and Places, a cross-sectional study of people's perceptions of how their neighborhood impacted their daily lives and actions and vice versa. Data were collected in Atlanta, Georgia. Participants (*n* = 1,864, 817 of whom were women) were recruited from 80 census block groups, using both active community outreach strategies (based on ethnographic information and interviews done with key informants) and passive recruitment methods. The “active community outreach” strategies used in this study utilized a best-practices approach for the inclusion of research participants from hidden populations, and they were undertaken by trained professional staff with specific expertise in working in difficult-to-reach communities, building trust among members of the target community, engaging potentially interested persons in the study and its research objectives, screening these persons for eligibility, and providing potential respondents with all informed consent-related information and procedures. The “passive” recruitment strategies primarily focused on posting flyers at a wide range of community settings frequented by members of the target population, including telephone poles in the targeted communities, message boards in local-area social service agencies and community health providers, retail stores and commercial outlets, message boards inside of religious institutions located in the targeted communities, and the offices of various community-based organizations. Active outreach accounted for approximately 80% of the persons who eventually enrolled in the study, with the passive recruitment methods accounting for the other 20%.* Post hoc *analysis of the data revealed no discernible differences in the demographic characteristics of persons who came into the study via active versus passive outreach efforts.

The census block groups chosen for inclusion in the study were selected based on neighborhood structural characteristics as reported in the 2000 US Census Data and based on data from the Atlanta Police Department. Consistent with the study's conceptual model and previous research findings [18, 19], key neighborhood structural characteristics for inclusion were (1) the percentage of household incomes that were reported to be more than 20% above or below the federal poverty level, (2) the percentage of adults who had not completed high school or its equivalent, (3) the percentage of female-headed households, (4) the percentage of people who were unemployed or not in the labor force, (5) the percentage of one-unit housing structures, (6) the percentage of owner-occupied households, and (7) the percentage of vacant housing. Within the selected census block groups, the sampling frame was designed to ensure sufficient variability by gender and age (specifically, persons who were under the age of 35 and those aged 35 or older, to facilitate analytical comparisons based on younger versus older adults).

In order to be considered eligible for participation, respondents had to self-identify as African American or Black, be at least 18 years of age, and have lived in the same neighborhood or census block group continuously for at least one year. People were considered ineligible for the study if they (1) were in an institutional setting at the time of recruitment, (2) were homeless at any time during the preceding year (because this would have run counter to the stable residency eligibility requirement, just described), (3) were intoxicated at the time of consent or interview, or (4) displayed signs of cognitive impairment at the time of consent or interview.

Computer-assisted structured interviews (CASI) were conducted with eligible persons in a private office that was located at a field site centrally located in one of the catchment areas. The survey covered information about people's demographic characteristics, psychological and psychosocial characteristics, substance use history, sexual activity, criminal justice involvement, support network, and neighborhood perceptions. On average, interviews lasted approximately 90 minutes. The Emory University Institutional Review Board approved the study protocol.

### 2.2. Measures

The main outcome variable used in this paper is a scale assessing the extent to which alcohol-related problems were experienced during the preceding year. The scale was comprised of 10 items, each indicating whether the experience in question occurred “never,” “a few times,” “quite a lot,” or “often” during the preceding year. For the purpose of determining the number of drinking-related problems, each item was recoded to indicate having “never happened” or “ever happened” during the previous year. Constituent items included (1) problems with one's family because of one's alcohol use, (2), problems with one's friends because of one's drinking, (3) legal problems resulting from alcohol (ab)use, (4) physical fights as a result of one's drinking, (5) having wanted to quit or cut down on one's drinking, (6) trying to hide one's drinking from others, (7) receiving complaints from others as a result of one's alcohol (ab)use, (8) having experienced cravings for alcohol, (9) having experienced withdrawal symptoms when unable to drink, and (10) having consumed more alcohol than one wanted to. The scale was found to be reliable (Kuder-Richardson_20_ = 0.81).

For the analyses associated with Research Question #2 (i.e., the factors associated with incurring a larger number of alcohol-related problems) and Research Question #3 (i.e., evidence of potentiating, or syndemic, effects among the key predictors of drinking problems), independent variables were included from each of the four domains shown in Figure 1 conceptual model.* Sociodemographic characteristics* used in these analyses included the following:* age* (ages of 18–29 versus ages of 30+),* relationship status* (married or “involved” in a relationship versus not married or not “involved”),* educational attainment* (did not complete high school or its equivalent versus at least a complete high school graduation or its equivalent),* employment status* (unemployed versus any employment),* sexual orientation* (lesbian or bisexual versus heterosexual),* religiosity* (continuous measure), and two measures of* health/medical insurance* (any versus no coverage during the preceding year; coverage for all 12 months versus 0–11 months of the preceding year).

*Childhood maltreatment experiences* were assessed using six scale measures derived from Bernstein and Fink's [20]* Childhood Trauma Questionnaire*. All items asked respondents about their experiences prior to age 18 and included sexual abuse (Cronbach's alpha = 0.95), physical abuse (Cronbach's alpha = 0.81), emotional abuse (Cronbach's alpha = 0.83), physical neglect (Cronbach's alpha = 0.71), emotional neglect (Cronbach's alpha = 0.85), and overall amount of maltreatment (Cronbach's alpha = 0.93).

Five scales, all of which were developed by the present authors specifically for use in the People and Places study based on the preimplementation formative research, were used to assess* neighborhood characteristics*.* Perceived safety of one's neighborhood* was comprised of seven four-point ordinal items scored from “very unsafe” to “very safe” (Cronbach's alpha = 0.70).* The frequency of crime in the neighborhood* during the previous six months used three four-point ordinal items scored from “never” to “often” to compose the scale (Cronbach's alpha = 0.70).* The frequency of witnessing violence in one's neighborhood* during the previous year was comprised of seven four-point ordinal questions scored from “never” to “often” (Cronbach's alpha = 0.87).* The overall quality of life in the respondent's neighborhood* was constructed from eight five-point Likert items ranging from “strongly disagree” to “strongly agree” (Cronbach's alpha = 0.82).* The extent to which neighbors were involved positively in one another's lives* was constructed from five items, ordinally scored using four response options ranging from “never” to “often” (Cronbach's alpha = 0.79).

Three measures were used to assess* personal support network characteristics*.* The extent to which the respondent's friends were involved in criminal activities* was based on eight five-point ordinal items asking about the proportion of the friends who were criminally involved, ranging from “none” to “all of them” (Cronbach's alpha = 0.85).* The overall quality of the person's friendship relationships* was based on responses to seven items (Cronbach's alpha = 0.89). Each item asked about various relationship qualities and asked respondents to indicate on a four-point ordinal scale (ranging from “not important at all” to “very important”) how important each quality was to them in a friendship. The* size of the person's support network* was a continuous measure derived by summing the number of males and the number of females each respondent said that she could count on for emotional support, financial support, and/or practical support.

### 2.3. Statistical Analysis

Research Question #1, pertaining to the prevalence of alcohol use and drinking-related problems, was addressed with the use of descriptive statistics.

Research Question #2, which examined factors underlying alcohol problems in this population, was analyzed in a few successive steps. Initially, bivariate analyses were performed to determine which of the independent variables were associated with the number of alcohol problems experienced. Whenever the independent variable was dichotomous (e.g., sexual orientation, educational attainment), Student's *t*-tests were conducted. Whenever the independent variable was continuous in nature (e.g., perceived safety of one's neighborhood, amount of childhood maltreatment experienced), correlation coefficients were computed. Subsequently, items identified in the bivariate analyses as being related significantly (*p* < .05) or marginally (.10 > *p* > .05) to the number of drinking problems experienced were retained for entry into a multiple regression equation to determine which items were associated with the outcome measure when the effects of the other measures under consideration were taken into account. Both forward selection and backward elimination procedures were used to develop a fully reduced (or saturated) model containing only statistically significant predictors.

For Research Question #3, which focused on whether or not the data provided specific evidence of syndemic effects, a variety of two-way and three-way interaction effects were examined for the key independent variables. To keep our analysis focused here, we elected to examine three specific independent variables for evidence of possible syndemic effects: age (comparisons were made between people aged 18–29 and those aged 30+), childhood maltreatment status (comparisons were based on persons experiencing no or very low levels of childhood maltreatment (i.e., mean scores ranging from 0 to 0.49) versus those experiencing higher levels of maltreatment (i.e., mean scores ranging from 0.5 to 4.0)), and criminal involvement of one's friends (comparisons were based on people who reported having no friends who were criminally involved versus those who said that at least one of their friends was criminally involved). These three measures were chosen for these particular analyses because the distribution of the data was such that these classifications were likely to be the most robust statistically and because the multivariate data suggested that if syndemic-type effects were occurring, these three measures were the most likely candidates to illustrate the presence of such effects. Analysis of variance was used to compare the number of drinking-related problems based on all possible two-way interactions and the possible three-way interaction of these variables.* Post hoc* paired-comparisons tests were performed as well, to determine which subgroup(s) of respondents differed statistically from which other group(s) in terms of their drinking problems rates.

Throughout all of these analyses, results are reported as being statistically significant whenever *p* < .05.

## 3. Results

### 3.1. Sample Characteristics

Table 1 provides a summary of the characteristics of this all-female subsample of the People and Places study. Study participants were relatively evenly divided between those under the age of 30 (48.3%) and those aged 30 or older (51.7%), with the past-year-nondrinkers being significantly more likely than those who had at least one drink during that time to be under the age of 30 (*p* < .01). Most of the respondents (77.0%) either had not completed high school or its equivalent (38.6%) or had graduated from high school or had earned a general equivalency diploma with no further education beyond that point (38.4%). Women who consumed alcohol during the previous year were more likely than those who had not to have had at least some college training (*p* < .001). Employment status was similar for the past-year drinkers and the past-year abstainers, with the majority of both groups (68.6% and 74.1%, resp., or 69.8% overall) reporting being unemployed (*n.s.*). Relationship status was also similar for the two groups, with 60.0% of the past-year drinkers and 64.4% of the past-year abstainers (61.0% overall) saying that they were married or “involved” with someone (*n.s.*). Sexual orientation did not differ significantly for the two groups either, with 9.8% of the study participants self-identifying as lesbian or bisexual (*n.s.*).

### 3.2. Prevalence of Alcohol Use and Drinking Problems

The large majority of the women who had ever consumed an alcoholic beverage (89.7%) reported having consumed at least one drink of alcohol during the preceding year. Almost all of these women (94.2%) indicated also having consumed at least one alcoholic drink during the previous 90 days. Throughout all of the remainder of the Results, past-year abstainers are excluded, so that the focus is on alcohol-using women.

Experiencing drinking-related problems was the norm, not the exception, among those reporting recent alcohol use. About one-third (32.8%) of those reporting alcohol consumption during the preceding year reported incurring no alcohol-related problems. Approximately one-third of the past-year alcohol users (32.7%) said that they experienced 1 or 2 of the 10 drinking-related problems that they were asked about. The mean number of drinking-related problems experienced was 3.3 (SD = 2.3). As Table 2 shows, the most commonly experienced alcohol-related problem were wanting to quit drinking or, alternatively, to cut down on one's alcohol consumption (49.6%), craving a drink (27.8%), receiving complaints from others about one's drinking (24.6%), experiencing problems with family members as a result of one's drinking (21.0%), and getting into physical fights as a result of one's drinking (20.1%).

### 3.3. Predictors of Drinking Problems

Of the seven* sociodemographic characteristics* measures examined, only relationship status was found to be unrelated to the number of alcohol-related problems women experienced. Women under the age of 30 reported slightly more than half as many alcohol problems as their counterparts who were aged 30 or older (1.3 versus 2.6, *p* < .001). Women who had not completed high school or its equivalent experienced significantly more problems from their drinking compared to their peers who had, at a minimum, completed high school or its equivalent (*p* < .001). Women who self-identified as lesbian or bisexual reported significantly more alcohol problems than their heterosexual counterparts did (*p* < .001). Study participants who had no health or medical insurance during the preceding year experienced a larger number of drinking-related problems than their counterparts who had insurance for at least part of the year (*p* < .001); and those who were insured for the entire previous year reported lower rates of alcohol interference in their lives than did persons who were insured for anything less than the entire preceding 12 months (*p* < .001).

All six of the* childhood maltreatment* variables were found to be related strongly (all *p* < .001) to the extent to which women incurred drinking-related problems. In all instances, more abuse or neglect corresponded with a larger number of alcohol problems.

Four of the five* neighborhood characteristics* were linked with women's drinking problems (with the sole exception being the item assessing neighbors' involvement in one's life). A larger number of alcohol-related problems were reported by women who perceived their neighborhood to be unsafe (*p* < .001), lived in neighborhoods with higher rates of crime (*p* < .001), and witnessed violence taking place first-hand in their local neighborhood (*p* < .001) and those who said that their overall neighborhood quality of life was poorer (*p* < .001).

Finally, two of the three* personal support network* items were related to the extent of women's drinking problems. The more friends the women had who were criminally involved, the more alcohol problems the women themselves reported experiencing (*p* < .001). Moreover, the more people whom women felt they could rely upon for emotional, financial, and/or practical support, the less adversely affected they were by alcohol (*p* < .01).

When the statistically significant preceding items were entered into a multivariate equation together to determine which ones contributed significantly to the overall model when the influence of the others was taken into account, seven items were found to contribute uniquely and significantly to the overall prediction of the number of drinking-related problems women experienced (see Table 3). These were as follows: being aged 30 or older (*p* < .001), having had no health insurance during the preceding year (*p* < .01), having less than a high school graduation-level education (*p* < .01), self-identifying as lesbian or bisexual (*p* < .01), experiencing greater amounts of childhood maltreatment (*p* < .001), being more impulsive by nature (*p* < .01), perceiving one's neighborhood to be unsafe (*p* < .01), and having a larger number of criminally involved friends (*p* < .001). Together, these eight items explained 25.0% of the total variance in the number of drinking-related problems experienced.

### 3.4. Evidence of Syndemic Effects

Considerable evidence was found in support of the notion that syndemic-type effects among key independent variables influenced the extent to which women experienced drinking-related problems. Highly statistically significant main effects (*p* < .0001) were obtained for all three of the two-way interaction effects examined. In all three instances, all but one of the paired-comparisons were also statistically significant, which demonstrates that it was the unique combination—that is, the conjoint effects—of (1) age and friends' criminal involvement, (2) age and childhood maltreatment experiences, and (3) friends' criminal involvement and childhood maltreatment experiences that led certain subgroups to be at much higher-than-average risk for incurring drinking problems. Similarly, the three-way interaction of these same variables was also statistically significant (*p* < .0001), with 20 of the 28 paired-comparisons yielding significant differences.

**Figure 2:**
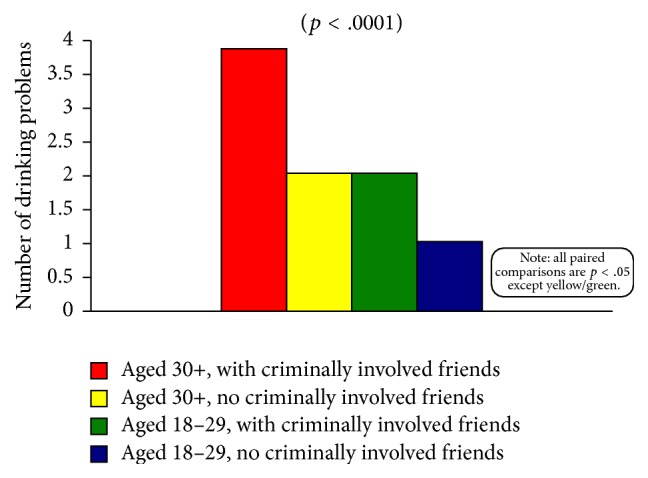
Number of drinking problems, by age and friends' criminality.

**Figure 3:**
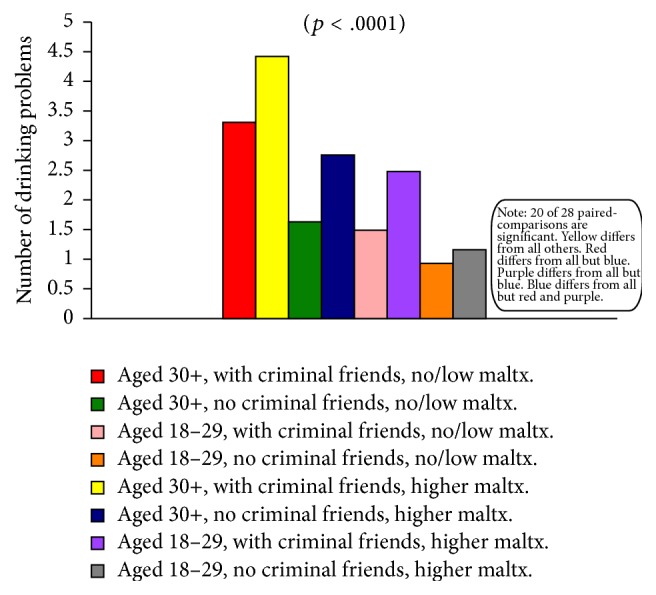
Number of drinking problems, by age group, friends' criminality, and childhood maltreatment.

Figure 2 (which presents the findings for the age × friends' criminal involvement analysis) is representative of the findings obtained for all three of the two-way comparisons; and Figure 3 depicts the findings obtained for the age × childhood maltreatment experiences × friends' criminal involvement analysis. Figure 2 clearly demonstrates that there is a heightening of effects when both age and friends' involvement in criminal activities are considered. Effects that are especially large are noticeable for the women aged 30 or older who have friends who are criminally involved versus women aged 18–29 who have no criminally involved friends. It is worth pointing out, too, that both of these particular groups also differed significantly from both of the others shown in Figure 2.

Figure 3 demonstrates that certain of the comparison groups experience far more or far fewer alcohol-related problems than others. As an excellent example, consider the second bar in the figure (shown in yellow), which represents women aged 30 or older with childhood maltreatment in their backgrounds and who had at least one criminally involved friend. These individuals incurred significantly more drinking problems than all other groups. Similarly, the first bar in the figure (shown in red) represents women aged 30 or older who had at least one criminally involved friend but whose backgrounds included very little or no childhood abuse or neglect. These women experienced significantly more alcohol problems than all other comparison groups with the exception of the fourth group (shown in dark blue), which happens to be comprised of women aged 30 or older with childhood maltreatment in their backgrounds but no criminally involved friends.

## 4. Discussion

### 4.1. Potential Limitations of the Research

The authors would like to acknowledge a few potential limitations of this research. First, it was conducted with a sample of African Americans residing in a major metropolitan area. Persons living in other environments that are less densely populated may not share the same life and community experiences as those living in urban areas such as the one where the present study was conducted. Additionally, this research was conducted in the American South. African Americans living in other parts of the country may have different socioecological experiences compared to the persons who participated in the People and Places study. The extent to which these geographic factors affected the present study's findings is not known and cannot be assessed with the available data.

Second, all data used in the People and Places study were based on uncorroborated self-reports. Therefore, the extent to which respondents underreported or overreported their involvement in various behaviors, such as alcohol consumption or drinking-related problems, is unknown. In all likelihood, the self-reported data can be trusted, as numerous authors have noted that persons in their research studies (which, like the present study, have included fairly large numbers of substance abusers and/or persons at risk for contracting or transmitting HIV) have provided accurate information about their behaviors [21, 22].

A third possible limitation pertains to recall bias. Respondents were asked to report about their beliefs, attitudes, and behaviors during the past 30 days, the past 90 days, and the past-year, depending upon the measure in question. These time frames were chosen specifically (1) to incorporate a large enough amount of time in the risk behavior questions' time frames so as to facilitate meaningful variability from person to person and (2) to minimize recall bias. The exact extent to which recall bias affected the data cannot be assessed although other researchers collecting data similar to that captured in this study have reported that recall bias is sufficiently minimal that its impact upon study findings is likely to be small [23], provided that the recall period for many types of frequency-counted behaviors does not exceed 90 days [24].

A fourth potential limitation pertains to the recruitment methods used for identifying study participants for the People and Places study. Both passive and active recruitment strategies were used in order to identify eligible respondents and to gain their involvement in the study. Passive recruitment approaches in particular are subject to self-selection bias because they rely upon people who are interested in the subject matter of the research to volunteer to participate in the study. As a result, certain individuals who find the research subject matter less salient to their lives may choose not to participate in studies such as People and Places. As with any research study relying upon voluntary participation (which means almost all research studies involving human subjects), the extent to which this type of bias occurred and affected the findings obtained in this study simply cannot be assessed. A comprehensive discussion of the various ways in which self-selection bias may and may not affect the quality of information obtained in community-based studies of alcohol abuse has been provided by Connors and Volk [25]; interested readers are encouraged to consult that article.

## 5. Conclusions

Despite these potential limitations, we believe that the present study has much to offer in terms of its insights into the issues surrounding alcohol abuse in the population in question. In this community-based sample of urban African American women in the southern United States, alcohol use was highly prevalent and, for most women, so too were drinking-related problems. More than two-thirds (67.2%) of the alcohol-using women in the study said that, during the preceding year, they had experienced at least one problem as a result of their drinking; and more than one-third (34.5%) of the alcohol-using women reported three or more of such problems during that time period. With the two most common problems of these drinking-related ones being a desire to quit drinking or, in the alternative, to cut down on one's drinking and experiencing alcohol-related cravings, there are strong indications of prevalent symptoms of alcohol dependence in this population. This suggests a need for alcohol(ism) treatment facilities that can offer recovery services to and be effective with African American women who reside in urban areas. Other authors have addressed the need to develop effective ways of facilitating substance abuse recovery among low-income, urban African American women [26–28]; and our findings are consistent with theirs and are concordant with their recommendations. Moreover, given that the other most commonly alcohol-related problems cited by the women in this study all pertained to the social and interpersonal disruptions that often result from repeated, excessive drinking, there is also considerable evidence in the present study for a need for alcohol intervention initiatives targeting alcohol-abusing African American women. For these women, full treatment and recovery-related services may not be needed. Rather, these persons might benefit from programs that can work with them to develop strategies to reduce their alcohol intake and/or to modify their alcohol consumption patterns so that alcohol (and problems ensuing from its use/abuse) can have less of an impact upon their lives in the future. Intervention approaches that emphasize reductions in alcohol consumption or alterations in drinking patterns, rather than entry into a substance abuse treatment program, have been recommended for African American women by other researchers [29]. Regardless of whether the approach taken is one of emphasizing recovery via treatment or harm reduction via intervention, it will be important that the approach taken is culturally sensitive to the needs of African American women—a point echoed by other authors as well [3, 30, 31].

The present study is also informative in its findings pertaining to the factors underlying drinking problems among southern, urban African American women. Seven such factors were identified in our multivariate analysis, and each merits a brief discussion. First, our analyses revealed that women aged 30 and older experienced significantly more problems as a result of their alcohol use compared to women aged 18 to 29. Various factors are likely to be relevant here. One of them is the simple matter of progression—that is, drinking problems and alcohol dependence progress and worsen as people continue to drink regularly, heavily, and/or abusively over time. Another factor that probably contributes to this finding, at least to some extent, is that the women at the lowest end of the adult age spectrum—namely, those aged 18 to 20—are unable to purchase alcohol legally or to consume it legally. This probably prevents some of them from having easy access to alcohol, which in turn leads to reduced alcohol-related problems among these younger women. Regardless of the underlying cause, the older women were more at risk for experiencing drinking-related problems than their younger counterparts, thereby leaving us with the recommendation of considering these older women to be a group in need of targeted intervention, education, and/or treatment with regard to alcohol-related matters. Other researchers have spoken about the need to offer age-targeted alcohol intervention programs [3, 32]. Our findings are consistent with those studies and their recommendations.

Second, our multivariate analysis revealed that women who had not had any health insurance during the previous year experienced approximately 50% more alcohol problems than their counterparts who were insured for part or all of that time. As of 2013 with the passage of the Affordable Care Act, all health insurance plans offer coverage for mental health counseling and/or substance abuse treatment. But not all adults have signed up for health insurance [33]. Having no medical insurance whatsoever may have led some uninsured women to forgo potentially beneficial counseling and/or recovery services that, ultimately, fostered their ongoing problems with alcohol. In their research based on young adults meeting the diagnostic criteria for substance dependence, Wu and Ringwalt [34] reported that 87% of the uninsured alcohol-dependent persons in their study received no substance abuse treatment services during the preceding year. The present study's finding highlights the importance of making health insurance available to and affordable for urban African American women as part of an ongoing effort to combat alcohol-related problems in this population.

Third, in this study, we learned that women who had not graduated from high school or earned a general equivalency diploma (GED) experienced approximately one-third more drinking-related problems than their better-educated counterparts. Based on this finding, it would be wise for alcohol education, alcohol intervention, and substance abuse treatment programs to target African American women with low levels of education. When preparing written materials for their target audience, educational and intervention initiatives may have to be particularly sensitive to the literacy levels of these women. Effective skills-building components of these programs may find it beneficial to incorporate information about the importance of earning a GED and to encourage women with lower levels of educational attainment to bolster their schooling.

Fourth, women who self-identified as lesbian or bisexual experienced more than 1.5 times as many alcohol problems as their heterosexual counterparts. Many studies have shown an increased risk for substance use and abuse among lesbians and bisexual women [35, 36]. The present research is consistent with those studies and expands upon them, by demonstrating that this finding also applies to a community sample of urban, southern African American women. Our finding highlights the need for specialized alcohol education, intervention, and treatment targeting women who consider themselves to be lesbian or bisexual. The need for such specialized services has been recognized by numerous agencies and providers, including the Substance Abuse and Mental Health Services Administration [37] and the National Association of Lesbian, Gay, Bisexual, and Transgender Addiction Professionals (see http://www.nalgap.org/ for more information about this organization). Excellent such programs exist around the United States, like the PRIDE Institute (Eden Prairie, MN), The Center (New York, NY), Morningside Recovery (Irvine, CA), Freedom Rings at Lakeview Health (Jacksonville, FL), among many others.

Fifth, the more childhood maltreatment that women experienced, the more alcohol-related problems that they incurred. This finding is consistent with numerous studies, which have shown an association between sexual, physical, and/or emotional abuse during the formative years and substance (ab)use problems in adulthood [38, 39], including research based on populations of African American women [40]. It underscores the need to consider previously maltreated women as a high-risk group in need of targeted intervention, as well as the importance of addressing past-abuse issues among affected women when they participate in substance abuse treatment programs.

Sixth, the more unsafe women perceived their neighborhood to be, the more alcohol problems they tended to experience. This finding is consistent with the main tenets of social disorganization theory (consult [41] for excellent readings on this subject). In recent years, there has been growing interest in the social-ecological factors that are associated with substance use and abuse and in the role that neighborhood factors may play in these problems [42]. Increasingly, evidence suggests that living in neighborhoods characterized by high rates of crime and violence, high rates of poverty, lower levels of neighborhood cohesion, and so forth elevates people's chances for developing substance abuse problems. For example, Davis and colleagues [27] noted that life stress (which very well may be heightened by living in a neighborhood that one perceives to be unsafe) is a causal mechanism for alcohol problems among women. Similarly, Karriker-Jaffe and colleagues [29] found that neighborhood disadvantage is associated with heavy drinking among African Americans—a finding that is closely akin to the one obtained in the present study. More research needs to be done to help us understand the various social-ecological forces that shape people's substance use and abuse behaviors, how it is that these forces influence drinking (and other drug-related) practices, and what factors mitigate the potential impact of neighborhood characteristics on substance use/abuse.

Seventh, drinking problems were more prevalent among people whose friends were criminally involved than they were among people whose friends were law abiding. Similar to the discussion (above) regarding social-ecological factors, here we have a finding that also pertains to environmental influences on individuals' drinking behaviors. In this instance, however, those influences are more direct and more personal, because they come in the form of friendships and how those friendships shape alcohol (ab)use practices. If the principles of social-ecological and social disorganization theories are applicable to the “feeling unsafe in one's neighborhood” finding discussed above, then the principles of social control theory, containment theory, and social bond theory (again, consult [41] for excellent readings on these topics) appear to be relevant to the “criminally involved friends” finding now being discussed. Ostensibly, these theories posit that behaviors are shaped based on people's relationships with others and the types of overt and covert influence they exert on them during their repeated interactions with them (social control); how individuals feel about and respond to the various expectations placed on them by other persons and institutions in their daily lives—family members, friends, their religious community, and neighborhood “elders” (containment)—will shape their behaviors; and the extent to which individuals are or feel connected to others in their daily lives and in their local neighborhood will determine the things that they will/will not do (social bond). Other researchers have examined these theorized influences and how they relate to substance (ab)use, typically finding that they are highly relevant to understanding alcohol and other drug-using behaviors [43, 44]. Interestingly, almost all of the published studies applying these theoretical models to alcohol and/or other drug-using behaviors have been conducted with adolescent populations. Consequently, less is known about how well these theories' tenets apply to adult populations, and less still is known about their applicability to adult populations of minority women. This, we believe, would be a fruitful avenue for future researchers to explore.

Finally, we would like to discuss our findings pertaining to the matter of whether or not the present research yielded evidence to support the notion of syndemic effects being operative with regard to the factors underlying drinking problems among urban African American women. It did. All of the two-way interaction analyses that we conducted not only demonstrated significant main effects but also demonstrated large intergroup differences when the combined effects of age and childhood maltreatment, or age and having criminally involved friends, or childhood maltreatment and having criminally involved friends were examined. The same was true for our analysis of the three-way interplay of age, childhood maltreatment experiences, and associating with criminally involved friends. These findings offer strong and consistent evidence of syndemic-type effects underlying the alcohol-related problems experienced by the southern, urban African American women we studied. As we mentioned earlier, although it has not been uncommon for syndemics-focused studies to incorporate substance use/abuse measures into their models [12–14], these studies emphasized illegal drug use/abuse or polydrug abuse rather than alcohol use/abuse specifically. Thus, the present study contributes to the scientific literature not only by documenting the applicability of syndemics theory to the field of alcohol studies but also by demonstrating its usefulness in elucidating the factors underlying drinking problems among African American women, which has been a population group to which this theoretical approach has been applied only rarely in the past.

Our findings, therefore, suggest two very important things: (1) the principles of syndemics theory may prove to be useful in the development of educational efforts and prevention/intervention initiatives striving to reduce the adverse impact of alcohol abuse. (2) Syndemics theory may be able to offer important and practical insights into the myriad factors underlying the health problems faced by African American women. On this basis, we recommend that future health educators, interventionists, and substance abuse treatment specialists working with African American women consider incorporating syndemics theory-based components into their alcohol education, intervention, and treatment endeavors. Our findings suggest that this may very well prove to be an effective approach for them to take.

## Figures and Tables

**Table 1:** Sample characteristics.

	No alcohol during past-year (*n* = 174)	Some alcohol during past-year (*n* = 643)
*Age group* ^*∗*^		
18–29	48.3	35.9
30–39	26.1	18.7
40–49	18.9	28.1
50 and older	16.7	17.3

*Educational attainment* ^*∗∗*^		
Less than high school graduation	31.0	40.6
High school graduation or equivalent	52.3	34.7
At least some college	16.7	24.7

*Employment status*		
Unemployed	74.1	68.6
Employed, part-time	9.2	14.2
Employed, full-time	8.1	8.1

*Relationship status*		
Not married or “involved”	35.6	40.0
Married or “involved”	64.4	60.0

*Sexual orientation*		
Heterosexual	93.1	89.4
Gay, lesbian, or bisexual	6.9	10.6

Please note that statistical differences between past-year alcohol users and nonusers are as follows:

^*∗*^ *p* < .01 and ^*∗∗*^ *p* < .001.

**Table 2:** Prevalence of alcohol-related problems.

Alcohol-related problem	Prevalence (%)
Wanted to quit or cut down on one's drinking	49.6
Consumed more alcohol than one wanted to	31.0
Experienced cravings for alcohol	27.8
Receiving complaints from others as a result of one's alcohol (ab)use	24.6
Problems with one's family because of one's alcohol use	21.0
Physical fights as a result of one's drinking	20.1
Problems with one's friends because of one's drinking	18.5
Trying to hide one's drinking from others	9.5
Legal problems resulting from alcohol (ab)use	9.2
Experiencing withdrawal symptoms when unable to drink	8.4

**Table 3:** Factors associated with the number of drinking problems women experienced.

Independent variable	*b*	*β*	*p* = |*x*|
Age = under 30	–1.27	0.25	<.001
Health insurance = none in previous year	0.54	0.11	.002
Educational attainment = less than high school	0.50	0.10	.004
Sexual orientation = lesbian or bisexual	0.75	0.09	.008
Amount of childhood maltreatment	0.52	0.16	<.001
Perceived level of neighborhood safety	–0.95	0.09	.007
Extent of friends' criminal involvement	1.00	0.24	<.001
